# Eggshell temperature during early and late incubation affects embryo and hatchling development in broiler chicks

**DOI:** 10.1016/j.psj.2022.102054

**Published:** 2022-07-15

**Authors:** Arda Sözcü, Aydın İpek, Henry van den Brand

**Affiliations:** ⁎ Bursa Uludağ University, Department of Animal Science, Bursa, Turkey; † Adaptation Physiology Group, Wageningen University and Research, 6700 AH, Wageningen, the Netherlands

**Keywords:** eggshell temperature, bone development, incubation, broiler, chick quality

## Abstract

This study aimed to investigate effects of eggshell temperature (**EST**) during early and late incubation on embryo and hatchling development of broiler chicks. A total of 720 eggs were randomly allocated to 3 treatment groups: control EST (37.8°C during the first 14 d and 36.8°C between d 15 and 21 of incubation), early high EST (as control, but 38.9${}^{\circ }$C between d 4 and 7), and late high EST treatment (37.8°C during the first 14 d and 38.2°C between d 15 and 21). At d 18 of incubation, the length of the femur, tibia, and metatarsus were found to be lower in the early high EST treatment than in both other treatment. Hatchability was higher in the early high and control EST treatment than in the late high EST treatment (Δ = 4.2% on average; *P* = 0.02), whereas the opposite was found for late term embryonic mortality (Δ = 4.0% on average; *P* = 0.02). Navel score was higher for the late high EST treatment than for the early high EST and control treatment (1.36 vs. 1.19 and 1.17, respectively; *P* < 0.001). At hatch, chick weight, and organ weights were lower in the late high EST treatment than in the control treatment, with the early high EST treatment in between. At hatch, most femur, tibia, and metatarsus characteristics were lower for the early high EST treatment compared to both other treatments. The same was found for tibia ash, Ca, and P concentrations. Blood ALP and P levels were higher in the control group than in both other treatment groups. It can be concluded that early high EST particularly affected bone development during incubation, whereas late high EST particularly resulted in a decline in hatchability and chick quality in broiler chicks.

## INTRODUCTION

Due to genetic selection and improved management strategies, broiler chicks have reached a high growth rate in the last decades, resulting in higher body weights at younger age (Zuidhof et al., 2014). These higher body weights at younger age are related to a higher incidence of leg problems, which cause negative effects on health and welfare (Weeks et al., 2000; Bessei, 2006), but also on performance (Bradshaw et al., 2002; Hashimoto et al., 2013). Leg problems can have a developmental, degenerative, or infectious origin (Van der Pol et al., 2014) and because leg bone development already starts during incubation, it has been suggested that leg problems during the rearing phase of broiler chicks might have their origin in suboptimal embryonic leg bone development (Oviedo-Rondon et al., 2008a, 2009a; Van der Pol et al., 2014; Güz et al., 2020). Cartilage formation starts during the first week of incubation (Nakane and Tsudzuki, 1999; Bellairs and Osmond, 2005; Atalgin and Kürtül, 2009), followed by a rapid ossification during the second week of incubation (Pechak et al., 1986a,b; Oviedo-Rondón et al., 2008b). This ossification reaches the highest speed just before hatching and continues in the first days post-hatching (Oviedo-Rondón et al., 2008b; Kürtül et al., 2009), resulting in a high relative length growth of long bones occur during the first week post-hatch (Applegate and Lilburn, 2002).

One of the most important factors affecting embryonic leg bone development is incubation temperature (Hammond et al., 2007; Oviedo-Rondón et al., 2008a,b, 2009a; Shim and Pesti, 2011; Van der Pol et al., 2014; Güz et al., 2020). Small deviations in incubation temperature (often expressed as eggshell temperature (Lourens et al., 2005; Ipek et al., 2014) from optimal (37.8°C; Lourens et al., 2005) might already affect embryonic leg bone development in different ways. EST deviations from control resulted in changes in tibia and femur growth (Hammond et al., 2007; Oviedo-Rondón et al., 2008b, 2009a; Van der Pol et al., 2014). Oviedo-Rondón et al. (2009b) found a lower leg bone weight when an EST of 38.9°C between embryonic d (**ED**) 18 to 21 was applied compared to an EST of 37.0 to 37.8°C. However, Oviedo-Rondón et al. (2008a) showed that femur weight was higher at hatching after an EST of 36.0 or 39.0°C than when an EST of 37.0 or 38.0°C was applied between ED 17 and hatch. Tibia length was higher at an EST of 38.0°C than at the other EST groups. Van der Pol et al. (2014) showed that a very high EST (39.4°C) between ED 0 and hatch resulted in the lowest tibia and metatarsus lengths (−3.1 to −8.4%) compared with a low (36.9°C), normal (37.8°C), and high (38.6°C) EST. The observed apparent inconsistencies among studies could be attributed to the used EST, the period of incubation in which EST was applied and other factors (genotype, breeder age, egg weight, etc.).

This study aimed to investigate effects of EST stimulations during early (38.9°C between ED 4 and 7) and late (38.2°C between ED 15 and 21) incubation on embryo development, with special emphasis on leg bone development. The hypothesis of this study is that leg bone development could be differentially changed by higher EST during different incubation periods, which might explain the ambiguous results described in literature.

## MATERIALS AND METHODS

All experimental protocols were approved by the ethical committee of Bursa Uludag University and practiced in accordance with the laws and regulations of Turkey (License Number 2015-13/07).

### Experimental Setup

A total of 720 eggs were obtained from a commercial Cobb 500 broiler breeder parent stock at 65 wk of age with an average egg weight of 71.3 g ± 0.8 (mean ± SE). After collection, eggs were stored at 16°C and 65% RH for 2 d and subsequently warmed to room temperature (22°C) for 8 h before setting. Eggs were numbered, weighed before incubation, and then randomly divided over 3 treatment groups: control eggshell temperature (**EST**), early term high EST (**early high**) and late term high EST (**late high**) treatment. In the control EST treatment, eggs were incubated at 37.8°C during the first 14 ED and at 36.8°C between ED 15 and 21. In the early high EST treatment, eggs were incubated at the same EST pattern as the control treatment, except that at ED 4–7, EST was increased to 38.9°C. In the late high EST treatment, eggs were incubated at 37.8°C during the first 14 ED and at 38.2°C between ED 15 and 21. Relative humidity was maintained between 55 and 60% throughout incubation. Eggs from the control EST and late high EST treatments (n = 3 trays per treatment, 80 eggs/tray) were incubated in the same and fully automated incubator (640 capacity egg setter, 6 trays: T640 I, Cimuka Inc., Ankara, Turkey) during the first 14 d. Eggs from the early high EST treatment were kept in another, comparable incubator (640 capacity egg setter, n = 3 trays per treatment; T640 I, Cimuka Inc.).

At ED 15, eggs were candled and transferred to hatching baskets (3 hatching baskets per treatment) and each hatching basket was considered as a replicate. Eggs in the control group and early high EST treatment were placed in the same hatcher (640 egg capacity hatcher, 8 trays: T640 H, Cimuka Inc.), and eggs in the late-term high EST treatment group were transferred into another comparable hatcher (640 egg capacity hatcher, 8 trays: T640 H, Cimuka Inc.).

### Measurements

At ED 18, 10 randomly selected embryos per EST treatment were euthanized by cervical dislocation, weighed and morphological measurements of femur, tibia, and metatarsus bones were performed. The embryos were excised from the extra embryonic membranes, carefully separated from the yolk sac and excessive fluid was dried off with absorbent paper. Residual yolk (**RY**) weight and yolk free body mass (**YFBM** = body weight – RY) were determined. Relative embryo and yolk weight were calculated as:

Relativeembryoweight(%)=(YFBM/eggweightatsetting)×100;

Relativeyolkweight(%)=(RY/eggweightatsetting)×100.

Embryo length was measured from the tip of the beak to the tip of the middle toe, excluding the nail by placing the embryo face down on a flat surface and straightening the right leg (Hill, 2001). Tibia, femur, and metatarsus (including cartilage) of both legs were frozen at −20°C until measurement of weight, length, and width.

From 498 h of incubation onward, the number of hatched chicks was recorded every 12 h, but chicks stayed in the incubator. At the end of the hatching period (510 h after start of incubation), chicks were classified as salable (clean, dry, and without deformities) or culls (splayed legs, unhealed navels, malformations etc.; Tona et al., 2004; Molenaar et al., 2011). The percentage of culled chicks was expressed as a percentage of fertile eggs. Hatchability of fertile eggs was expressed as a ratio between the number of hatched saleable chicks and the number of set fertile eggs. Unhatched eggs were opened to macroscopically determine fertility or embryonic mortality (early term mortality during the first week of incubation, middle term mortality between 8 and 18 d of incubation, late term mortality between 19 and 21 d of incubation). All hatched chicks were weighed, and navel condition was scored as 1 (a clean and closed navel), 2 (a black button or gap smaller than 2 mm), or 3 (a black button or gap larger than 2 mm; Molenaar et al., 2011).

Approximately 12 h after pulling, 10 randomly selected chicks per EST treatment was randomly sampled for chick quality parameters and organ weights. Chick body weight and length were measured by the same procedures as the embryonic measurements. Chicks were euthanized by cervical dislocation to determine RY weight, YFBM, and organ weight (heart, gizzard, liver, small intestine, spleen, thymus, and bursa of Fabricius). Relative weight of organs was expressed as a percentage of YFBM.

At hatch, blood sampling was performed by the punctuation of the jugular vein from 10 chicks in each treatment group. Samples were collected in heparinized tubes, centrifuged at 1,200 × *g* for 15 min and plasma was stored in vacutainer tubes. Plasma levels of alkaline phosphatase (**ALP**), Ca and P were determined, using a Roche autoanalyzer (Cobas 6000 series C501 module, Roche Diagnostic, Indianapolis, IN) and Roche kits.

To evaluate leg bone development at hatch, tibia, femur, and metatarsus of both legs (including cartilage) were removed and frozen at −20°C until measurements. After thawing, the bones were checked for any residue of soft tissues and then held at 22°C for 7 d to allow for drying. After drying, the bones were weighed (Model XB 4200C, Precisa Corp, Zurich, Switzerland), and bone length and bone width (at 50% of the bone length) were measured, using a caliper (Model CDN-20C, Mitutoyo Corp, Aurora, IL).

The relative weight of the tibia, femur, and metatarsus was calculated as ratio between bone weight and chick weight. Then, relative asymmetry for bone length was calculated with the formula given by Møller et al. (1999):

RA={|R−L|/[(R+L)/2]}×100

in which, RA means relative asymmetry of the left and right bone (%), R means length of the right bone (mm), L means length of the left bone (mm), and |R − L| means the absolute difference between R and L.

Breaking strength (N) for each bone obtained from embryos and chicks was determined by a 3-point bending test, using a fully computerized UTEST tensile and compression testing machine (Model 7014, UTEST Corp, Ankara, Turkey) that was fitted with a 250 kN load cell. The crosshead movement was at 10 mm/min.

The right tibia was ashed, using AOAC method 932.16 (AOAC International, 2005). Tibias were subjected to a temperature of 105°C for 6 h and then defatted with hexane in a Soxhlet apparatus (Model SER148, Şimşek Laborteknik, Ankara, Turkey) for 4 h. After the extraction of fat, the bones were dried in a forced-ventilated oven at 105°C for 16 h to determine the dry and defatted weights of tibias. Then, the bone samples were crushed and calcined in a muffle furnace at 600°C for 2 h to determine the ash content. Approximately 1 g of ash sample was then dissolved in 10 mL of HNO_3_ and 10 mL of HCl and boiled for 10 min. The sample was filtered and diluted into a 50 mL flask. After obtaining this solution, the calcium and phosphorus contents in the tibias were obtained through Optical Emission Spectrometry with an Inductively Coupled Plasma source (**ICP-OES**) (Optima 2100 DV, Perkin Elmer Inc, Shelton, CT).

### Statistical Analysis

All statistical analyses were performed with the statistical package Minitab 19 (Minitab 19.1 Version, Minitab Inc, State College, PA). Hatchability, embryonic mortalities, percentage of cull and salable chicks, chick hatching weight, and navel score were analyzed by one-way ANOVA, with the model:

(1) Y=μ+EST+e,

Where Y = dependent variable, µ = overall mean, EST = treatment group (control, early high EST, late high EST), e = residual error. Egg tray was used as the experimental unit.

Embryo and leg bone development at ED 18 and chick quality parameters, organ growth, leg bone, and blood characteristics at hatch were analyzed by a one-way ANOVA, using model 1. Embryo or chick was considered as the experimental unit.

Analyses for relative data (expressed as percentage) were conducted after square root of arc sine transformation of the data. Significant differences among treatment means were determined by the Duncan's multiple range test. Data are presented as LSmeans ± pooled SEM. In all cases, a difference was considered significant at *P* ≤ 0.05.

## RESULTS

Effects of EST treatments during incubation on embryo development and leg bone characteristics at ED 18 are presented in Table 1. No EST treatment effects were found for yolk and relative yolk weight and embryo and relative embryo weight. Embryo length was higher in the control treatment than in the early high EST treatment (Δ = 3.9 mm; *P* = 0.01), with the late high EST treatment in between. Length of femur (Δ = 1.28 mm on average; *P* = 0.02), tibia (Δ = 2.22 mm on average; *P* < 0.01), and metatarsus (Δ = 1.69 mm on average; *P* = 0.002) were lower in embryos of the early high EST treatment compared to both other treatments. Additionally, relative weight of the metatarsus was lower in the early high EST treatment than in both other treatments (Δ = 0.03% on average; *P* = 0.03).

**Table 1:** Effects of different eggshell temperature (EST) treatments during incubation on embryo development and leg bone morphological characteristics at day 18 of incubation (n = 10 embryos/treatment group; LSmeans ± SEM).

Items	EST treatments^1^			SEM	*P*-value
	Early	Control	Late		
Yolk weight (g)	15.2	16.3	16.2	1.43	0.19
Relative yolk weight (%)^2^	21.3	22.9	22.9	2.11	0.17
Embryo weight (g)	31.5	31.5	31.0	1.61	0.67
Relative embryo weight (%)	44.1	44.4	43.5	2.17	0.65
Embryo length (cm)	15.9^b^	16.3^a^	16.1^ab^	0.55	0.71
Femur					
Weight (g)	0.064	0.069	0.066	0.008	0.39
Relative weight (%)	0.20	0.22	0.21	0.03	0.39
Length (mm)	14.787^b^	16.172^a^	15.959^a^	0.836	0.02
Width (mm)	1.617	1.637	1.632	0.120	0.92
Tibia					
Weight (g)	0.084	0.090	0.091	0.014	0.50
Relative weight (%)	0.27	0.29	0.29	0.04	0.37
Length (mm)	20.462^b^	22.948^a^	22.417^a^	1.029	0.001
Width (mm)	1.551	1.665	1.625	0.114	0.09
Metatarsus					
Weight (g)	0.053	0.063	0.062	0.009	0.05
Relative weight (%)	0.17^b^	0.20^a^	0.20^a^	0.02	0.02
Length (mm)	15.617^b^	17.195^a^	16.815^a^	0.944	0.002
Width (mm)	1.345	1.435	1.419	0.094	0.09

1 Control = 37.8°C during the first 14 d and 36.8°C between d 15 and 21 of incubation; Early = as control, but 38.9°C between d 4 and 7 of incubation; Late = 37.8°C during the first 14 days and at 38.2°C between d 15 and 21 of incubation.

2 Relative yolk weight (%) = (RY / egg weight at setting) × 100.

a–b LSmeans within a row lacking a common superscript differ (*P* ≤ 0.05).

Effects of EST treatments during incubation on hatchability, embryo mortality, and chick quality of all chicks at hatch are presented in Table 2. The late high EST treatment resulted in a lower hatchability (Δ = 4.3% on average; *P* = 0.02), a higher late term embryonic mortality (Δ = 4.0% on average; *P* = 0.02) and a higher (worse) navel score (Δ = 0.18 on average; *P* < 0.001) compared to both other groups. A higher percentage of navel score 2 (23.9%) and 3 (6.2%) was observed in the late term high EST group compared to both other groups (*P* < 0.01). The percentage of culled chick, and chick hatching weight did not differ between EST treatments. Navel status of the chicks was worse in the late term high EST treatment than in the control and early term high EST treatments (1.36 vs. 1.17 and 1.19, *P* < 0.01).

**Table 2:** Effects of different eggshell temperature (EST) treatments during incubation on hatchability, embryo mortality and chick quality characteristics of all hatched chickens (n = 3 trays/treatment group, 80 eggs/tray, LSmeans ± SEM).

Items	EST treatments^1^			SEM	*P*-value
	Early	Control	Late		
Hatchability of fertile eggs (%)	89.4^a^	89.9^a^	85.4^b^	1.4	0.02
Early embryonic mortality (%)^2^	4.0	3.5	3.5	1.0	0.82
Mid embryonic mortality (%)^2^	2.6	2.6	3.1	1.1	0.84
Late embryonic mortality (%)^2^	4.0^b^	4.0^b^	8.0^a^	1.3	0.02
Culled chick (%)^3^	1.97	1.95	3.63	0.87	0.09
Chick hatching weight (g)	49.7	51.0	49.0	1.02	0.13
Navel score^4^	1.19^b^	1.17^b^	1.36^a^	0.03	<0.001

1 Control = 37.8°C during the first 14 d and 36.8°C between d 15 and 21 of incubation; Early = as control, but 38.9˚C between d 4 and 7 of incubation; Late = 37.8°C during the first 14 d and at 38.2°C between days 15 and 21 of incubation.

2 Early term mortality during the first week of incubation, middle term mortality between 8 and 18 d of incubation, late term mortality between 19 and 21 d of incubation.

3 Culled chicks were defined as chicks with splayed legs, unhealed navels, malformations etc. (Tona et al. 2004; Molenaar et al. 2011).

4 Navel scored as 1: a clean and closed navel, 2 = a black button or gap smaller than 2 mm, 3 = a black button or gap larger than 2 mm (Molenaar et al., 2011).

a–b LSmeans within a row lacking a common superscript differ (*P* ≤ 0.05).

Effects of EST treatments during incubation on chick quality parameters and organ weights at hatch of 10 chicks per treatment group are presented in Table 3. Residual yolk weight (Δ = 1.24 g on average; *P* < 0.001) and percentage (Δ = 2.78% on average; *P* < 0.001) were higher in the late high EST treatment than in both other treatments. Chick body weight (Δ = 1.73 g; *P* = 0.04) and YFBM (Δ = 3.12 g; P < 0.001) were lower in the late high EST treatment than in the control treatment, with the early high EST treatment in between. Spleen weight (Δ = 0.08 g on average; *P* = 0.01) and percentage (Δ = 0.017% on average; *P* = 0.04) were lower in the late high EST treatment compared to both other treatments. Weight of heart (Δ = 0.06 g; *P* = 0.007), liver (Δ = 0.15 g; *P* = 0.02), small intestine (Δ = 0.31 g; *P* = 0.02), and Bursa of Fabricius (Δ = 0.02 g; *P* = 0.007) and percentage of Bursa of Fabricius (Δ = 0.04%; *P* = 0.05) were lower in the late high EST treatment than in the control treatment, with the early high EST treatment in between.

**Table 3:** Effects of different eggshell temperature (EST) treatments during incubation on chick quality parameters and organ growth at hatch (n = 10 chicks/treatment group, LSmeans ± SEM).

Items	EST treatments^1^			SEM	*P*-value
	Early	Control	Late		
Chick quality parameters					
Chick body weight (g)	49.35^ab^	50.59^a^	48.86^b^	1.45	0.04
Residual yolk weight (g)	5.90^b^	5.55^b^	6.96^a^	0.59	<0.001
Residual yolk weight (%)^2^	11.95^b^	10.98^b^	14.24^a^	1.18	<0.001
Yolk-free body mass (g)	43.45^ab^	45.03^a^	41.91^b^	1.44	<0.001
Body length (mm)	19.19^ab^	20.02^a^	18.26^b^	0.58	<0.001
Organ weight (g)					
Heart	0.41^ab^	0.45^a^	0.39^b^	0.04	0.007
Gizzard	2.20	2.22	2.08	0.19	0.20
Liver	1.30^ab^	1.35^a^	1.20^b^	0.12	0.03
Small intestine	2.15^ab^	2.26^a^	1.95^b^	0.21	0.01
Spleen	0.026^a^	0.027^a^	0.018^b^	0.006	0.01
Thymus	0.03	0.04	0.03	0.01	0.17
Bursa of Fabricius	0.05^ab^	0.06^a^	0.04^b^	0.01	0.007
Relative weight (% of YFBM)					
Heart	0.94	0.99	0.93	0.09	0.24
Gizzard	5.06	4.93	4.96	0.45	0.79
Liver	2.99	3.00	2.86	0.32	0.55
Small intestine	4.95	5.02	4.65	0.56	0.32
Spleen	0.060^a^	0.060^a^	0.043^b^	0.014	0.03
Thymus	0.07	0.08	0.07	0.03	0.45
Bursa of Fabricius	0.12^ab^	0.13^a^	0.09^b^	0.02	0.041

1 Control = 37.8°C during the first 14 d and 36.8°C between d 15 and 21 of incubation; Early = as control, but 38.9˚C between d 4 and 7 of incubation; Late = 37.8°C during the first 14 d and at 38.2°C between d 15 and 21 of incubation.

2 Residual yolk weight was calculated as ratio between residual yolk weight and chick body weight.

a–b LSmeans within a row lacking a common superscript differ (*P* ≤ 0.05).

Effects of EST treatments during incubation on leg bone morphological and mechanical traits at hatch are presented in Table 4. The early high EST treatment resulted in lower femur weight, relative weight, length and breaking strength, tibia weight, relative weight, length and width, and metatarsus width and breaking strength than both other groups. The early high EST treatment also resulted in lower femur width and metatarsus width than the control treatment, with the late high EST treatment in between. Tibia relative asymmetry was lower in the control treatment than in the late high EST treatment and the opposite was found for metatarsus relative asymmetry, with the early high EST treatment in between. Tibia breaking strength was higher in the late high EST treatment than in both other treatments.

**Table 4:** Effects of different eggshell temperature (EST) treatments during incubation on leg bone morphological and mechanical traits at hatch (n = 10 chicks/treatment group, LSmeans ± SEM).

Items	EST treatments^1^			SEM	*P*-value
	Early	Control	Late		
Femur					
Weight (g)	0.17^b^	0.26^a^	0.25^a^	0.02	<0.001
Relative weight (%)^2^	0.39^b^	0.58^a^	0.60^a^	0.05	<0.001
Length (mm)	20.83^b^	22.58^a^	22.15^a^	0.79	<0.001
Width (mm)	2.91^b^	3.30^a^	3.26^ab^	0.34	0.03
Relative asymmetry (%)^3^	0.70	0.35	0.38	0.49	0.24
Breaking strength (N)	31.69^b^	37.20^a^	36.33^a^	1.74	<0.001
Tibia					
Weight (g)	0.34^b^	0.44^a^	0.43^a^	0.03	<0.001
Relative weight (%)^2^	0.69^b^	0.87^a^	0.89^a^	0.06	<0.001
Length (mm)	29.53^b^	31.27^a^	31.59^a^	1.24	0.002
Width (mm)	3.23^b^	3.87^a^	3.75^a^	0.32	<0.001
Relative asymmetry (%)^3^	0.27^ab^	0.11^b^	0.51^a^	0.28	0.01
Breaking strength (N)	23.03^b^	23.81^b^	25.77^a^	1.15	<0.001
Metatarsus					
Weight (g)	0.31	0.33	0.32	0.03	0.40
Relative weight (%)^2^	0.64	0.65	0.65	0.06	0.83
Length (mm)	22.34	23.23	22.50	1.16	0.20
Width (mm)	3.49^b^	3.87^a^	3.73^ab^	0.29	0.02
Relative asymmetry (%)^3^	0.84^ab^	0.93^a^	0.52^b^	0.35	0.03
Breaking strength (N)	21.14^b^	25.48^a^	24.46^a^	2.36	0.001

1 Control = 37.8°C during the first 14 d and 36.8°C between d 15 and 21 of incubation; Early = as control, but 38.9°C between d 4 and 7 of incubation; Late = 37.8°C during the first 14 d and at 38.2°C between d 15 and 21 of incubation.

2 The relative weight of the tibia, femur, and metatarsus was calculated as ratio between bone weight and chick weight.

3 The relative asymmetry was calculated with the formula given by Møller et al. (1999): RA = {|R − L|/[(R + L)/2]} × 100; in which, RA = relative asymmetry of the left and right bone (%), R = length, depth, or width of the right bone (mm), L = length, depth, or width of the left bone (mm), and |R − L| = the absolute difference between R and L.

a–b LSmeans within a row lacking a common superscript differ (*P* ≤ 0.05).

Effects of EST treatments during incubation on tibia ash and mineral content, and blood ALP, Ca, and P at hatch are presented in Table 5. Tibia ash content was highest in the control treatment, followed by the late high EST and early high EST treatment. Ca (Δ = 1.54% on average; *P* < 0.001) and P (Δ = 1.29% on average; *P* = 0.001) concentration were lower in the early high EST treatment than in both other treatments. Blood ALP concentration was highest in the control treatment, followed by the early high EST treatment and late high EST treatment. Blood P concentration was higher in the control treatment than in both other treatments (Δ = 0.9 mg/dL on average; *P* = 0.007).

**Table 5:** Effects of different eggshell temperature (EST) treatments during incubation on tibia and blood characteristics at hatch (n = 10 chicks/treatment group, LSmeans ± SEM).

Items	EST treatments^1^			SEM	*P*-value
	Early	Control	Late		
Tibia characteristics					
Ash (%)	30.34^c^	35.91^a^	33.97^b^	1.49	<0.001
Ca (%)	9.23^b^	11.06^a^	10.48^a^	0.56	<0.001
P (%)	4.18^b^	5.73^a^	5.21^a^	0.82	0.001
Blood characteristics					
ALP (IU/l)^2^	2979.7^b^	3089.3^a^	2835.6^c^	61.5	<0.001
Ca (mg/dL)	9.48	9.70	9.71	0.65	0.67
P (mg/dL)	5.57^b^	6.59^a^	5.70^b^	0.72	0.007

1 Control = 37.8°C during the first 14 d and 36.8°C between d 15 and 21 of incubation; Early = as control, but 38.9°C between d 4 and 7 of incubation; Late = 37.8°C during the first 14 d and at 38.2°C between d 15 and 21 of incubation.

2 ALP, alkaline phosphatase.

a–b LSmeans within a row lacking a common superscript differ (*P* ≤ 0.05).

## DISCUSSION

The aim of this experiment was to investigate effects of high EST stimulation during early or late incubation on embryo development, hatchability, organ growth, and chick quality in broilers, with special emphasis on leg bone development. The results suggested that at one hand a high EST in early incubation had a detrimental effect on leg bone development during the embryonic phase and at hatch. At the other hand, a high EST in late incubation caused a decline in hatchability due to a higher late term embryonic mortality and a poorer chick quality at hatch, but did not affect leg bone development.

### General Chick Quality

Many studies showed that yolk absorption and broiler embryo development and growth are sensitive to incubation temperature (Lourens et al., 2005, 2007; Molenaar et al., 2011; Maatjens et al., 2014, 2016; Ipek et al., 2014, 2015; Wijnen et al., 2021). The majority of studies focused on higher EST in late incubation, whereas effects of increased EST in early incubation are less investigated. Both hatchability and chick quality can be affected by EST during incubation (Joseph et al., 2006; Meijerhof, 2009; Willemsen et al., 2010; Ipek et al., 2014, 2015). The current study showed that a high EST in the last wk of incubation increased late term embryonic mortality, decreased hatchability, and resulted in a worse navel score. These results are supported by Ipek et al. (2015), who reported that a higher EST of 38.4 to 39.0°C between ED 19 and 21 applied for only 3 h daily or constantly caused a serious decline in hatchability (93.6% and 90.1% vs. 97.8%) and an increase in the percentage of culled chick (3.9% and 1.8% vs. 0.4%) compared to the control eggs in broilers. Avsar et al. (2022) showed a higher hatchability when eggs were incubated at control EST (37.8°C through 21 d of incubation, 94.9%) or at a higher EST of 38.6°C between 1 and 3 d of incubation and thereafter applied at the control EST, 95.6%), compared to the other high EST treatments (an EST of 38.6°C between 1 and 6 d of incubation and thereafter applied at the control EST, 89.1%, and an EST of 38.6°C between 3 and 6 days of incubation, before and thereafter at the control EST, 90.7%).

One-day old chick quality is important for a good start of the broiler's life and for subsequent growth performance and health (Meijerhof, 2009). Current findings showed that high EST treatment in late incubation caused deterioration in chick quality parameters as chick body weight, residual yolk weight, and yolk free body mass. These findings are in accordance with Leksrisompong et al. (2007), Willemsen et al. (2010), Molenaar et al. (2011), and Ipek et al. (2015). The observed differences among the treatment groups could be explained by previous findings reported by Nangsuay et al. (2017). Embryos incubated at high EST (38.9°C) at later stage of incubation require more oxygen for nutrient metabolism to meet the energy requirements, but oxygen availability is restricted by the eggshell conductance (Tazawa et al., 1988; Whittow and Tazawa, 1991). Consequently, nutrient metabolism and yolk nutrient absorption is reduced, resulting in lower YFBM and higher RY weight. Embryos in the control and early high EST treatment group may use the yolk sac for development of muscle and the organs (Willemsen et al., 2010).

In addition to poorer chick quality parameters, chicks obtained by late term high EST treatment had a lower weight of immune organs as spleen and bursa of Fabricius at hatch, which might have consequences for later life immune competence (Wijnen et al., 2020).

### Bone Development

The skeletal formation of the embryo starts during the first days of the incubation period, with formation of chondrocytes in cartilage forming hind limbs and forelimbs around ED 5 and 6 (Bellairs and Osmond, 2005). Consequently, the first week of incubation is a sensitive period in which incubation temperature could have a potential delaying effect on cell differentiation. During incubation, bones continue to ossify and growth and, consequently increase in strength and stiffness. The growth rate of the bones reaches the highest level at the end of the last wk of incubation, which means that leg incubation temperature also might influence bone development in this phase (Applegate and Lilburn, 2002; Yair et al., 2012; Van der Pol et al., 2014; Güz et al., 2020). Consequently, the EST fluctuations in both early and late incubation might affect embryonic bone development and in turn might affect post-hatch performance and the incidence of leg problems of broilers (Toscano et al., 2013).

Current results indicated that the growth of leg bones of broiler chicks at hatch can be affected by higher EST, but the size of the effect on bone development depended on the moment of EST application (early or late incubation). Additionally different leg bones (femur, tibia, and metatarsus) were affected to varying degrees when embryos were exposed to higher EST. Particularly the early high EST treatment had negative effects on femur, tibia, and metatarsus traits, whereas the effects of the late high EST treatment were limited.

The current study showed that effects of high EST in early or late incubation clearly differ. The early high EST treatment particularly affected leg bone development, but had less effect on general chick quality at hatch, whereas the opposite was found for the late high EST treatment. That a high EST might affect bone development is supported by for instance Hammond et al. (2007), Oviedo-Rondón et al. (2009a), Van der Pol et al. (2014), and Güz et al. (2020). They showed that EST fluctuations from the optimum temperature range affected bone development, depending on the incubation period in which EST was applied and the level of the EST. This shows that effects of EST on leg bone develop strongly depends on the developmental stage of the embryo in which EST manipulations are applied (Hammond et al., 2007; Oviedo-Rondón et al., 2009b).

The observed more severe effects of the early high EST treatment on leg bone development than late term high EST treatment might be related to the fact that in early incubation the framework of bones is laid down and in later incubation only ossification is taking place. During the development of the framework, when processes are speeded up by a high EST, more mechanisms can go wrong, resulting in retarded bone development at hatch. The development of tibia bone in broiler chicks could be measured by bone ash and mineral content, and subsequently bone breaking strength (Onyango et al., 2003; Shim et al., 2012). Therefore, bone ash and mineral content could act as an indicator of bone mineralization (Shim et al., 2012). Early high EST treatment decreased the ash, Ca, and P content of the tibia at hatch, which parallels the findings in morphological and mechanical bone traits, such as breaking strength. The higher levels of ash, Ca, and P levels of tibia in the control and late term high EST treatment groups provided bone stiffness. Shao et al. (2019) found a positive relationship between the P level of the tibia and tibia ash content, and breaking strength.

Recently, studies showed that ALP is a significant biochemical marker and could be used as indicator for skeletal development in vertebrates and, also in poultry (Gade et al., 2011; Semenenko et al., 2021). Moreover, age-related variations in ALP activity could be seen in poultry species due to bone formation processes (Li et al., 2014). Indeed, the activity of ALP in bones could be predicted the rate of bone mineralization of the leg and wing bones (Semenenko et al., 2021). These researchers noted that the ALP levels could show an increment associated with increasing of osteoblastic activity, primarily of the lower limbs. In our study, a high EST in both early and late incubation resulted in lower blood ALP and P concentrations, suggesting that bone calcification might be retarded due to high EST in both phases of incubation. This finding is supported by another study performed by Kamanli et al. (2021) who reported a significant decline blood ALP level of laying chicks at hatch exposed to eggshell temperature of 38.5°C (high), compared to the other chicks exposed to eggshell temperature of 36.9°C (low) and eggshell temperature of 37.5°C (control) between 7 and 21 d of incubation period.

ALP is involved in the bone calcification process (Li et al., 2014) and consequently blood ALP levels and, also Ca and P levels were investigated at hatch. A high EST in both early and late incubation resulted in lower blood ALP and P concentrations, suggesting that bone calcification might be retarded due to high EST in both phases of incubation. Between ED 12 and 16, the activity of ALP in embryo tibia bones increases for mineralization (Li et al., 2014). When comparing the ALP levels between treatment groups, it can be hypothesized that the lowest level of ALP in the late high EST treatment is related to the completion of bone mineralization and growth due to an accelerated hatching process which could be resulted in less time for bone mineralization. The higher levels of ALP and P in the control group can be related with continuing of the active period of the bone mineralization at hatch. This could be attributed to the higher activity of ALP in the plasma during hatching and the first days of post-hatch period, indicating the bone metabolism and formation of chicks (Shao et al., 2019).

In nature, there is no flawless bilateral symmetry for body parts in living organisms in real sense (Srivastava et al., 2018). Accordingly, under normal conditions bone asymmetry with a low degree could be acceptable in healthy 1-day old chicks. For example, there is a small asymmetry between right and left tibia bones of healthy chicks. However, during early incubation period, especially critically period for skeletal formation of chick embryo, some effecting factors could have some detrimental effects on bone forming process (Hammond et al., 2007; Oviedo-Rondón et al., 2009b; van der Pol et al., 2014). In this study, to understand the structural bone development of broiler chicks exposed to high EST stimulation during early or late incubation period, the relative asymmetry was assessed among the treatment groups. This could be meaningful indicator a susceptibility of fast-growing broiler against bone anomalies, such as tibial dyschondroplasia, rickets, and femoral head necrosis, especially during post-hatch growing period (Dinev, 2012).

The current findings remarkably showed that EST treatments during any time of incubation period resulted in significant fluctuations for relative asymmetry values for tibia and metatarsus in the treatment groups. In a previous study performed by Oviedo-Rondón et al. (2009b), a significant increase was observed in relative asymmetry of tibia bone in the 1-day-old chicks exposed to low incubation temperature (36.7°C) compared to the high eggshell temperature (39°C between d 18 and 21 of incubation) and control eggshell temperature (37.5°C during incubation period).

In conclusion, this study showed that a high EST during different phases of the incubation period effect different physiological pathways involved in embryo development. Early high EST was particularly detrimental for bone development, whereas late high EST was retarding general chick quality.

## DISCLOSURES

I hereby declare, on behalf of all authors, that we do not have any conflict of interest in relation to the study described in the manuscript “Eggshell temperature during early and late incubation affects embryo and hatchling development in broiler chicks”. The study described in this manuscript is executed according to the code of conduct of Uludag University and Wageningen University.
